# Association Between Perceived Stress and Fatigue Severity in Patients With Chronic Illnesses

**DOI:** 10.7759/cureus.86444

**Published:** 2025-06-20

**Authors:** Vaisnavy Govindasamy, Marium Nadeem Khan, Zeeshan Khalid Awan, Abali Wandala, Muhammad Bilal Akbar, Aaleen Kamran, Mohamed Attian, Warda Shahbaz, Abeeha Mahmood, Meera Al Shamsi, Zill-e- Rukh Fatima Ameer

**Affiliations:** 1 Medicine, South Tees NHS Trust, Middlesbrough, GBR; 2 Medicine, Shifa College of Medicine, Islamabad, PAK; 3 College of Medicine, MindWave Research Center, Islamabad, PAK; 4 Interventional Cardiology, Mission Vascular and Vein Institute, Mission, USA; 5 Faculty of Health Science-Medicine, Universidad Adventista del Plata, Paraná, ARG; 6 Psychiatry, Tallaght University Hospital, Dublin, IRL; 7 Pathology, Foundation University Medical College, Islamabad, PAK; 8 Surgery, Milton Keynes University Hospital, Milton Keynes, GBR; 9 Medicine, University Hospital Southampton, NHS Foundation Trust, Southampton, GBR; 10 Medicine, Combined Military Medical College and Hospital, Lahore, PAK; 11 Internal Medicine, Zayed Higher Organization for People of Determination, Abu Dhabi, ARE

**Keywords:** chronic illness, fatigue severity, perceived stress, psychological well-being, sleep duration

## Abstract

Background: Stress and fatigue are two of the most important contributing factors to the declining physical and mental well-being of patients suffering from chronic illnesses. This research aims to identify the direct relationship between stress and fatigue to improve treatment guidelines and patient care.

Methodology: This observational study was conducted from February to April 2025 in Islamabad, Pakistan. The study included adults over 18 years of age, individuals who provided consent, and those diagnosed with chronic illnesses such as diabetes, cardiovascular disease, autoimmune disorders, or cancer. A questionnaire was used to collect data comprising the Perceived Stress Scale and Fatigue Severity Scale. Analysis was performed through IBM SPSS Statistics for Windows, Version 26 (Released 2018; IBM Corp., Armonk, New York, United States).

Results: Around 181 participants (47%) were male, 165 (43%) were female, and 40 individuals (10%) preferred not to disclose their gender. The largest age group was 26-35 years, with 240 individuals (62%), followed by 69 participants (18%) aged 36-45 years. The most commonly reported condition was hypertension, affecting 125 individuals (32%), followed by cardiovascular disease in 114 participants (29%) and diabetes in 57 (15%). A statistically significant moderate positive correlation between stress and fatigue (r = 0.481, p < 0.01) was obtained.

Conclusion: A strong positive correlation exists between perceived stress and fatigue severity among individuals with chronic diseases, influenced by factors like sleep duration and time since diagnosis.

## Introduction

Chronic illnesses are diseases that persist for an individual’s entire lifetime from the time of diagnosis and include conditions such as rheumatoid arthritis, Crohn’s disease, diabetes mellitus, carcinomas, demyelinating conditions of the central nervous system, such as multiple sclerosis, and various sequelae of chronic liver and kidney disease. All of these conditions are growing in incidence and have a major impact on the sufferer’s physical and mental well-being. Research shows that women suffering from rheumatoid diseases experience various debilitating symptoms that limit their mobility and augment stress [1]. Similarly, cancer patients experience high levels of stress and depression during their treatment regimens due to excessive hospital visits, resulting in a major compromise on their quality of life and survival instincts [2].

Multimorbidity, defined as the co-existence of multiple conditions, has been estimated as a major cause of depressive symptoms in patients as well as their relatives [3]. In fact, with the burgeoning increase in the world population, the incidence of chronic illnesses, particularly those secondary to gene mutations, is also increasing, thus presenting a significant public health problem that requires critical attention. Among the major effects of chronic illnesses, fatigue is the most commonly reported one and is mostly defined as a feeling of extreme exhaustion and burnout that is not associated with the level of physical activity and rarely disappears with adequate rest.

It adds to patients’ level of stress and makes them irritable, resulting in poor motivation to deal with their disease symptoms and a lack of social alertness [4]. Fatigue is extremely prevalent in the subset of patients suffering from chronic diseases and is reported by 40-74% of these patients [5], highlighting the fact that this symptom must not be overlooked and should be adequately accounted for in clinical practice. On the contrary, perceived stress is defined as an individual’s ability to identify the severity of the experienced stress and decide whether it is bearable or not. Perceived stress is a subjective measure reflecting how individuals assess and cope with the stress they experience [6]. Stress plays a major role in the development of psychosomatic disorders such as depression, which is one of the leading causes of mental disability all around the world [7]. Moreover, stress is one of the major contributors to the increased perception of chronic pain in individuals with devastating lifelong diseases [8,9].

When considering the relationship between perceived stress and fatigue severity in individuals suffering from chronic illnesses, it must be noted that many pathophysiological as well as biological mechanisms are involved in establishing a link between them. It is well-known that the experience of feeling stressed releases certain hormones from the hypothalamic-pituitary-adrenal (HPA) axis, resulting in a wide array of symptoms such as feeling low, unmotivated, and lacking physical energy to perform everyday tasks [10]. The imbalance and the complex interplay of various hormones with each other result in feelings of fatigue, resulting in the lack of energy and mentally exhaustive symptoms of depression and anxiety, along with social withdrawal [11].

Despite there being a growing body of research identifying the risk factors for the development of stress and fatigue in sufferers of chronic diseases, there exists little evidence regarding the direct relationship between these two factors and how they might contribute directly towards the compromise in the quality of life of individuals living with debilitating chronic illnesses. The purpose of this study is to identify the direct relationship between these two contributing factors and to assess how they interact with each other, thus underlining major clinical areas that need improvement in order to add some semblance of peace in the lives of patients suffering from chronic conditions. Identifying the complex relationship between these factors may lead to new guidelines aimed at improving care delivery for these patients and enhancing their quality of life.

Rationale

Chronic illnesses significantly compromise the physical and psychological well-being of patients. Among the most commonly reported symptoms, fatigue and stress are often under-recognized yet highly impactful. Despite the increasing prevalence of these conditions, there is limited data on the direct association between perceived stress and fatigue severity in chronically ill populations.

This study is crucial in understanding how psychological stress exacerbates fatigue and how different factors, such as sleep, illness duration, and treatment status, may contribute to this relationship. By establishing a clear link, healthcare professionals can better tailor holistic and psychosocial interventions, such as stress management strategies, into standard treatment plans. The findings aim to enhance patient care and improve the quality of life for those battling chronic illnesses.

Primary objective

To examine the relationship between perceived stress and fatigue severity in individuals with chronic illnesses. This includes identifying the strength and direction of the association to better understand how psychological stress contributes to physical symptoms.

Secondary objectives

To evaluate the influence of lifestyle factors, such as smoking, alcohol use, and sleep duration levels, on perceived stress and fatigue; to compare stress and fatigue severity across different chronic illness groups (e.g., diabetes, hypertension, cancer); to assess whether perceived stress predicts fatigue severity using regression analysis; and to explore the relationship between treatment types and illness duration with stress and fatigue outcomes.

## Materials and methods

Study design

An observational, cross-sectional study was carried out between February and April 2025 at the Ahmed Medical Complex, Islamabad, Pakistan. Researchers focused on people who had been diagnosed with chronic illnesses. People took part in the study from outpatient sections of three large hospitals and one community health clinic in Islamabad. Potential participants were approached through flyers, spoken invitations, and suggestions from doctors.

Before any data were collected, every participant gave written permission. Responses were made anonymous, and the data was kept in locked files that only the research team members could open and check. MindWave Research Center, Islamabad’s Institutional Review Board, granted ethical approval for the study using the number IRB-2025-0023.

Sample size and selection criteria

The sample size formula for an infinite population was used to calculate the sample size for this study: \[n = \frac{Z^2 \cdot p (1 - p)}{d^2}\]

Where Z is a statistic for the level of confidence interval, p is the expected prevalence or proportion, and d is the margin of error or precision. Thus, for 95% confidence, the value of Z is 1.96, and the margin of error is 0.05. For proportion, the value giving the maximum possible size was used because of no previous data. Thus, 0.5 was used as a proportion. Using these values, the calculated sample size was 386 [12].

A well-defined selection criterion was applied to include and exclude participants. Participants were included if they were aged 18 years or older; patients had chronic diseases including diabetes, hypertension, cardiovascular disease, respiratory illness (e.g., asthma, chronic obstructive pulmonary disease (COPD)), autoimmune disorder, and cancer; and participants who gave consent to take part in the study. Similarly, participants were excluded if they were under 18 or did not have a diagnosed chronic illness.

Data collection

Data were collected using non-probability convenience sampling. A structured questionnaire was administered, either in printed form or electronically via tablets, depending on participant preference. Trained data collectors were present to assist with completion when needed. The questionnaire comprised four sections. The first section captured demographic information, including age, gender, education level, occupation, income, and urban or rural residence (Appendix 1). The second section collected general health and lifestyle details, such as type and duration of chronic illness, treatment modality, sleep duration, and habits like smoking and alcohol consumption (Appendix 2).

Perceived Stress Scale

The third section used the Perceived Stress Scale (PSS), a 10-item instrument developed by Levenstein et al. (1993) [13], to assess perceived stress levels (Appendix 3). Responses were scored on a five-point Likert scale ranging from zero (never) to four (very often). Positively worded items (items four, five, seven, and eight) were reverse-scored. The total score ranged from zero to 40 and was categorized into low (zero-13), moderate (14-26), or high (27-40) stress. PSS showed good internal consistency with Cronbach's α = 0.78-0.9.

Fatigue Severity Scale

The fourth part consisted of the Fatigue Severity Scale (FSS), which was created by Krupp et al. in 1989 (Appendix 4) [14]. There were nine items, and participants had to indicate on a seven-point Likert scale how much they agreed with the statements. Scores of four or higher out of all the items meant the person had significant fatigue. FSS also showed good reliability with Cronbach's α = 0.88.

Permission to use both the PSS and the FSS was obtained from the original publishers. The key characteristics and psychometric properties of the PSS and FSS are summarized in Table 1.

**Table 1:** Summary of the Perceived Stress Scale and Fatigue Severity Scale used in the study

Scale	Perceived Stress Scale (PSS)	Fatigue Severity Scale (FSS)
Developed by	Levenstein et al. (1993) [13]	Krupp et al. (1989) [14]
Purpose	To assess perceived stress levels	To assess the severity of fatigue
Total Items	10 items	9 items
Response Format	Five-point Likert scale (zero = never, four = very often)	Seven-point Likert scale (one = strongly disagree, seven = strongly agree)
Scoring	Total score: 0-40	Total score: 1-7 per item, score ≥ 4 on any item = significant fatigue
Reliability	Cronbach's α = 0.78-0.91	Cronbach's α = 0.88
Validity	Strong construct validity with other stress measures	High correlation with other fatigue scales and clinical relevance

Statistical analysis

The statistical analysis of the data was carried out with IBM SPSS Statistics for Windows, Version 26 (Released 2018; IBM Corp., Armonk, New York, United States). To check if the data follows a normal distribution, the Kolmogorov-Smirnov and Shapiro-Wilk tests were conducted on the data. The study results revealed that both the PSS and FSS score distributions were much different from normal (p < 0.05 for both tests). For this reason, non-parametric tests were carried out as well. Percentages and frequencies were obtained using descriptive tests. Correlation between fatigue and stress using the FSS and PSS, respectively, was done with the Pearson correlation test. Independent t-tests and ANOVA were used to compare the means of different study variables in the context of stress and fatigue. A linear regression analysis was performed to assess perceived stress as a predictor of fatigue severity. The chi-square test was used to assess the association between type and duration of chronic illness with average sleep duration per night. Similarly, the association between the current treatment type and two variables: duration of chronic illness and average sleep duration per night, was done using the chi-square test. Significance throughout the study was established with a p-value below 0.05.

Ethical considerations

This study took into account strict ethical procedures to safeguard the rights and welfare of the subjects. All data about participants were handled with the highest level of confidentiality and kept in locked files and accessible only to members of the research team. Particular attention was paid to the protection of vulnerable persons, including persons with chronic diseases, who should have been adequately informed of their rights, and participation should have been voluntary. There was also the maintenance of data integrity, where relevant measures were put to address any missing or incomplete data ethically. These were the precautions that were observed to ensure that the best ethical practices were maintained during the entire research process.

## Results

Table 2 presents the demographic and clinical characteristics of the 386 participants included in the study. The largest age group was 26-35 years, with 240 individuals (62%), followed by 69 participants (18%) aged 36-45. A total of 60 individuals (15%) were aged 18-25 years, while only 14 (4%) and three (1%) were aged 46-55 and 56-65 years, respectively, indicating a predominantly younger to middle-aged sample. In terms of gender, 181 participants (47%) were male, 165 (43%) were female, and 40 individuals (10%) preferred not to disclose their gender. Regarding marital status, 164 participants (43%) were married, 110 (28%) were single, 91 (24%) were divorced, and 21 (5%) were widowed. Educational attainment varied, with 145 participants (38%) having completed primary education, 128 (33%) having secondary education, and 75 individuals (19%) having no formal education. Only 29 participants (8%) had completed college or graduate-level education, and nine (2%) had postgraduate degrees. Employment status showed that 163 participants (42%) were unemployed, while 106 (28%) were retired, 79 (20%) were employed, and 38 (10%) were students.

**Table 2:** Demographic characteristics of participants (N = 386) f: frequency; %: percentage; COPD: chronic obstructive pulmonary disease

Variable	f	%
Age	-	-
18-25 years	60	15
26–35 years	240	62
36–45 years	69	18
46–55 years	14	4
56-65 years	3	1
Gender	-	-
Male	181	47
Female	165	43
Prefer not to say	40	10
Marital status	-	-
Single	110	28
Married	164	43
Divorced	91	24
Widowed	21	5
Educational level	-	-
No formal education	75	19
Primary	145	38
Secondary	128	33
College/graduate	29	8
Postgraduate	9	2
Employment status	-	-
Student	38	10
Employed	79	20
Unemployed	163	42
Retired	106	28
Type of chronic illness	-	-
Diabetes	57	15
Hypertension	125	32
Cardiovascular disease	114	29
Respiratory illness (e.g., asthma, COPD)	54	14
Autoimmune disorder	18	5
Cancer	18	5
Duration of chronic illness	-	-
Less than 1 year	121	31
1-3 years	136	35
4-6 years	99	26
7-10 years	25	7
More than 10 years	5	1
Current treatment type	-	-
Medication only	76	20
Therapy/counselling only	127	33
Both medication and therapy	130	34
None	53	14
Do you smoke?	-	-
Yes	266	69
No	120	31
Do you consume alcohol?	-	-
Yes	234	61
No	152	39
Average sleep duration per night	-	-
Less than 4 hours	74	19
4-6 hours	140	36
7-8 hours	129	33
More than 8 hours	43	11

Regarding chronic illnesses, the most commonly reported condition was hypertension, affecting 125 individuals (32%), followed by cardiovascular disease in 114 participants (29%) and diabetes in 57 (15%). Respiratory illnesses such as asthma or COPD were reported by 54 individuals (14%), while autoimmune disorders and cancer were each reported by 18 participants (5%). The majority of participants had been diagnosed within the last one to three years (136 participants, 35%), followed by 121 (31%) with illnesses lasting less than one year, 99 (26%) for four to six years, 25 (7%) for seven to 10 years, and five (1%) for more than 10 years. In terms of current treatment, 130 individuals (34%) were receiving both medication and therapy, 127 (33%) reported therapy or counseling only, and 76 (20%) were using medication alone. Fifty-three participants (14%) were not receiving treatment. Lifestyle data showed a high prevalence of smoking, with 266 participants (69%) identifying as current smokers, while 120 (31%) did not smoke. Similarly, 234 individuals (61%) consumed alcohol, whereas 152 (39%) reported no alcohol consumption.

Sleep duration data revealed that 140 participants (36%) reported sleeping four to six hours per night, 129 (33%) slept seven to eight hours, and 43 (11%) reported more than eight hours of sleep. Notably, 74 participants (19%) reported sleeping fewer than four hours per night, indicating possible risks related to chronic sleep deprivation in this sample.

Table 3 shows that the data in both the PSS and FSS groups are not normally distributed, according to the findings of the Kolmogorov-Smirnov and Shapiro-Wilk tests. On the PSS, the Kolmogorov-Smirnov test came back with a significant p-value going below 0.001, and the Shapiro-Wilk test gave a p-value of 0.018. Based on the tests, it looks like the data follows a pattern other than the typical normal one. In the same way, both Kolmogorov-Smirnov and Shapiro-Wilk tests for the FSS indicated a p-value that is less than 0.001, confirming that the data is not normally distributed. As both scales have p-values that are less than 0.05 in both tests, we reject the idea that the data are normally distributed. So, for the sake of statistical analysis, it is better to look into non-parametric tests since the data does not meet the normality assumption required by parametric tests.

**Table 3:** Normality test to check equal distribution among the Perceived Stress Scale and the Fatigue Severity Scale df: degree of freedom; parametric test: p>0.05; non-parametric test: p≤0.05

Variables	Kolmogorov-Smirnov			Shapiro-Wilk		
	Statistic	df	p	Statistic	df	p
Perceived Stress Scale	0.067	386	<0.001	0.991	386	0.018
Fatigue Severity Scale	0.082	386	<0.001	0.984	386	<0.001

Table 4 presents the intercorrelation between perceived stress and fatigue severity, showing a statistically significant moderate positive correlation between the PSS and the FSS (r = 0.481, p < 0.01). This indicates that participants with higher levels of perceived stress also reported greater fatigue severity, suggesting a parallel increase in psychological and physical symptom burden.

**Table 4:** Intercorrelations between the study variables *: p<0.05, **: p<0.001 considered significant; correlation: Pearson correlation

Variable	Perceived Stress Scale	Fatigue Severity Scale
Perceived Stress Scale	-	0.481^**^
Fatigue Severity Scale	0.481^**^	-

Table 5 presents the comparison of perceived stress and fatigue severity scores based on smoking status. Smokers (n = 266) reported a mean PSS score of 28.9 ± 3.8, while nonsmokers (n = 120) had a slightly higher mean score of 29.1 ± 3.7. The difference was statistically significant (t = -0.177, p = 0.006), but the 95% confidence interval (- -0.895 to 0.747) and the negligible t-value suggest the effect size is minimal and likely not clinically meaningful. For fatigue severity, smokers had a higher mean FSS score of 31.2 ± 4.9 compared to 30.3 ± 5.4 in nonsmokers. This difference was statistically significant (t = 1.590, p = 0.001), with a confidence interval ranging from -0.209 to 1.985, indicating a modest elevation in fatigue levels among smokers.

**Table 5:** Comparison among variables (smoking status) M: mean; SD: standard deviation; LL: lower limit; UL: upper limit; Cl: confidence interval; independent t-test, p < 0.05 considered statistically significant.

Variable	Yes (N = 266); M±S.D	No (N = 120); M±S.D	t	P	Cl 95% LL	UL	Cohen’s D
Perceived Stress Scale	28.9±3.8	29.1±3.7	-0.177	0.006	-0.895	0.747	-
Fatigue Severity Scale	31.2±4.9	30.3±5.4	1.590	0.001	-0.209	1.985	-

Table 6 presents the comparison of perceived stress and fatigue severity scores based on alcohol consumption. Participants who reported alcohol use (n = 234) had a mean PSS score of 29.0 ± 3.6, which was identical to the non-alcohol group (n = 152; 29.0 ± 4.0). The difference was statistically non-significant (t = -0.044, p = 0.964), with a 95% confidence interval ranging from -0.796 to 0.760, indicating no meaningful association between alcohol use and perceived stress. Similarly, fatigue severity scores did not differ significantly between the groups. The alcohol group had a mean FSS score of 30.7 ± 5.1, while the non-alcohol group scored 31.3 ± 4.9 (t = -1.093, p = 0.275; 95% CI: -1.621 to 0.462), suggesting that alcohol consumption was not significantly related to fatigue severity in this sample.

**Table 6:** Comparison among variables (alcohol consumption) M: mean; SD: standard deviation; LL: lower limit; UL: upper limit; CI: confidence interval; independent t-test; p < 0.05 considered statistically significant

Variable	Yes (N = 234); M±S.D	No (N = 152); M±S.D	t	P	Cl 95% LL	UL	Cohen’s D
Perceived Stress Scale	29.0±3.6	29.0±4.0	-0.044	0.964	-0.796	0.760	-
Fatigue Severity Scale	30.7±5.1	31.3±4.9	-1.093	0.275	-1.621	0.462	-

Table 7 compares perceived stress and fatigue severity scores across different chronic illnesses. The highest PSS scores were observed among participants with autoimmune disorders (M = 30.5, SD = 3.6) and diabetes (M = 30.1, SD = 3.4). In contrast, the lowest stress levels were seen in cardiovascular disease and cancer participants (M = 28.4). Although mean differences were noted, the overall variation was not statistically robust, as indicated by a non-significant F-value (F(5,380) = 2.200), suggesting that perceived stress did not significantly differ across illness types.

**Table 7:** Comparison of variables (type of chronic illness) M: mean; S.D: standard deviation; F: F-ratio; η2: effect size; one-way ANOVA; p < 0.05 considered statistically significant

Variable	Diabetes (N = 57); M±S.D	Hypertension (N = 125); M±S.D	Cardiovascular disease (N = 114); M±S.D	Respiratory illness (N = 54); M±S.D	Autoimmune disorder (N = 18); M±S.D	Cancer (N = 18); M±S.D	p	F (5,380)	η2
Perceived Stress Scale	30.1±3.4	28.9±3.7	28.4±3.9	29.1±4.1	30.5±3.6	28.4±3.3	0.05	2.200	0.028
Fatigue Severity Scale	30.3±4.9	30.2±5.4	31.7±4.7	30.8±4.7	31.9±5.9	31.9±5.8	0.02	1.404	0.018

The FSS scores were relatively consistent across groups for fatigue severity. The highest fatigue levels were reported by individuals with autoimmune disorders and cancer (both M = 31.9, SD ≈ 5.8), while those with hypertension (M = 30.2, SD = 5.4) and diabetes (M = 30.3, SD = 4.9) had slightly lower fatigue scores. However, the ANOVA test showed no statistically significant differences (F(5,380) = 1.404), indicating that fatigue severity did not vary meaningfully based on this sample's type of chronic illness.

Table 8 compares perceived stress and fatigue severity across average sleep duration groups. PSS scores showed minimal variation between groups, with the highest mean score reported by participants sleeping more than eight hours per night (M = 29.8, SD = 3.6) and the lowest among those sleeping four to six hours (M = 28.6, SD = 3.7). However, the difference was not statistically significant (F(3,382) = 1.284), indicating that perceived stress was not meaningfully associated with sleep duration.

**Table 8:** Comparison of variables (average sleep duration per night) M: mean; S.D: standard deviation; F: F-ratio; η2: effect size; one-way ANOVA; p < 0.05 considered statistically significant

Variable	Less than 4 hours (N = 74); M±S.D	4-6 hours (N = 140); M±S.D	7-8 hours (N = 129); M±S.D	More than 8 hours (N = 43); M±S.D	p	F (3,382)	η2
Perceived Stress Scale	28.9±3.9	28.6±3.7	29.2±3.9	29.8±3.6	0.028	1.284	0.010
Fatigue Severity Scale	29.5±4.7	30.9±5.0	30.9±5.4	33.2±4.3	0.003	4.765	0.036

In contrast, a significant difference was observed in fatigue severity scores across sleep duration categories (F(3,382) = 4.765). Participants who slept more than eight hours reported the highest fatigue levels (M = 33.2, SD = 4.3), while those with less than four hours had the lowest fatigue scores (M = 29.5, SD = 4.7). The pattern suggests a non-linear relationship, with short and long sleep durations associated with elevated fatigue, although the effect size was modest.

Table 9 presents a linear regression analysis examining perceived stress as a predictor of fatigue severity. The model shows that the PSS was a statistically significant predictor of FSS scores (β = 0.481, p = 0.003). The unstandardized coefficient (B = 0.064) indicates that fatigue severity increased by 0.064 points on average for each one-point increase in perceived stress. However, the 95% confidence interval for the coefficient (-0.070 to 0.199) crosses zero, suggesting some uncertainty around the estimate's precision despite statistical significance. The constant term was also significant (B = 29.041, p < 0.001), representing the expected FSS score when perceived stress is zero. The model indicates a statistically significant but small positive association between perceived stress and fatigue severity, with a standardized coefficient (β) of 0.481.

**Table 9:** Linear regression analysis predicting Fatigue Severity Scale (FSS) scores using Perceived Stress Scale (PSS) B: coefficient; S.E: standard error; β: standardized coefficient; LL: lower limit; UL: upper limit; Cl: confidence interval; p < 0.05 considered significant

Variable	B	95% Cl		S.E	β	P
-	-	LL	UL	-	-	-
Constant	29.041	25.11	32.97	1.999	-	<0.001
Perceived Stress Scale	0.064	-0.070	0.199	0.068	0.481	0.003

**Figure 1:**
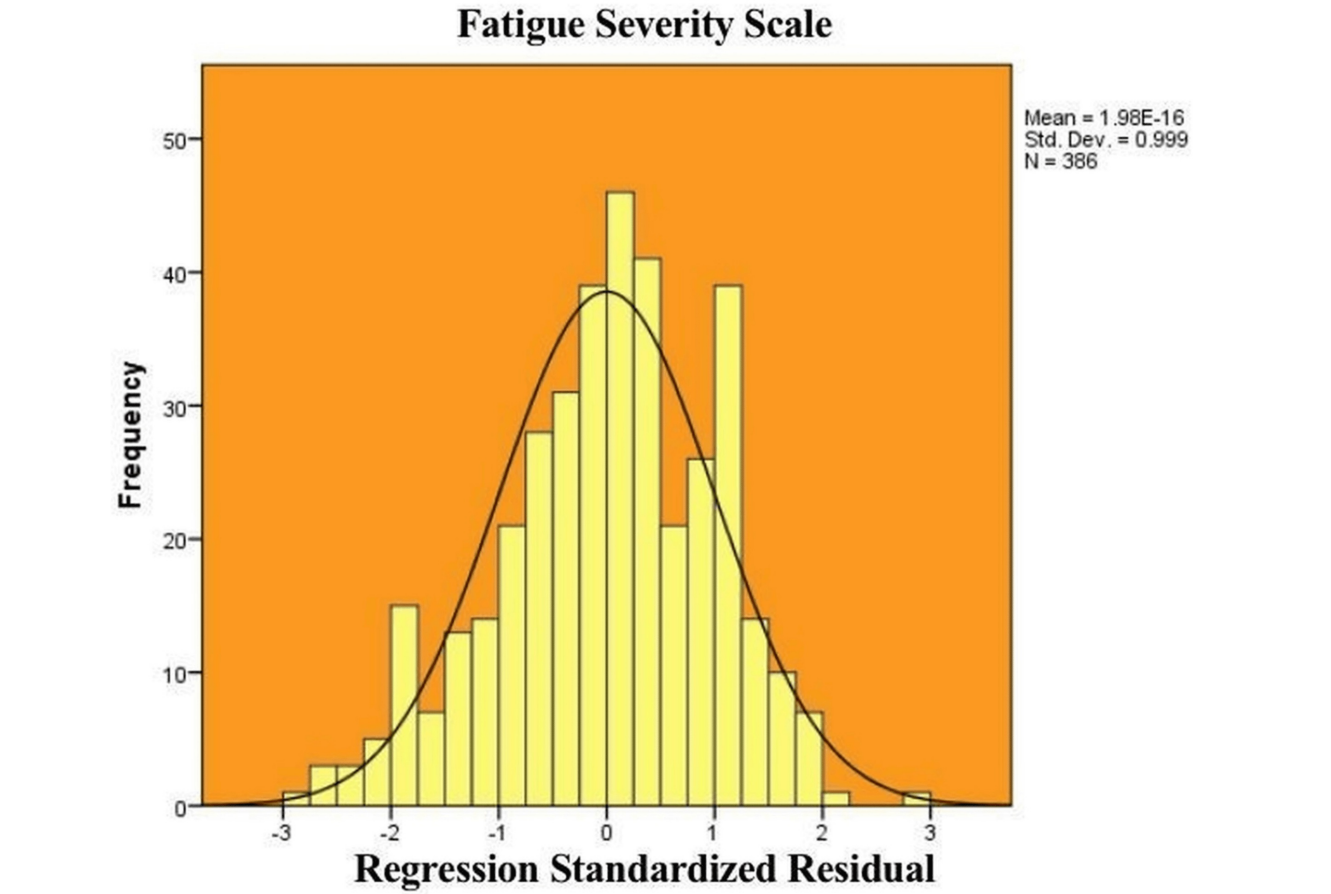
Histogram of regression standardised residuals for Fatigue Severity Scale

Figure 1 presents a histogram of the regression standardized residuals for the FSS. The distribution appears normal, with residuals centered around zero and most values falling within ±3 standard deviations. The bell-shaped curve and the symmetry of the bars indicate that the assumption of normality has been met, supporting the validity of the linear regression model. The mean residual value is zero, and the standard deviation is close to one, confirming that the residuals are appropriately distributed for parametric analysis.

Table 10 presents the association between type and duration of chronic illness with average sleep duration per night using the chi-square test. A statistically significant association was found between the type of chronic illness and duration of illness (χ² = 27.3, p = 0.006). Participants with hypertension and cardiovascular disease were more likely to have been diagnosed within the past one to three years (n = 41 and 38, respectively), while those with autoimmune disorders and cancer showed greater variability, including a small number reporting illness lasting seven to 10 years or more. This suggests that certain chronic conditions may present earlier or be detected more frequently in shorter time frames.

**Table 10:** Descriptive statistics of demographic variables (type of chronic illness, duration of chronic illness, and duration of sleep per night) f: frequency; %: percentage; p: level of significance; p-values calculated using the chi-square test; the significance level is set at p < 0.05

Variables	f	Less than 1 year	1-3 years	Duration of chronic illness (4-6 years)	7-10 years	More than 10 years	p	x^2^	Less than 4 hours	Duration of sleep per night (4-6 hours)	7-8 hours	More than 8 hours	p	x^2^
Type of chronic illness	-	-	-	-	-	-	0.006	27.3	-	-	-	-	0.395	15.8
Diabetes	57	18	21	15	3	0	-	-	14	21	17	5	-	-
Hypertension	125	51	41	28	5	0	-	-	25	50	37	13	-	-
Cardiovascular disease	114	32	38	32	9	3	-	-	21	38	42	13	-	-
Respiratory illness	54	14	21	14	4	1	-	-	10	12	25	7	-	-
Autoimmune disorder	18	1	11	3	3	0	-	-	2	8	5	3	-	-
Cancer	18	5	4	7	1	1	-	-	2	11	3	2	-	-

In contrast, the association between type of chronic illness and duration of sleep per night was not statistically significant (χ² = 15.8, p = 0.395). Participants with different chronic conditions reported similar distributions across sleep duration categories. For example, individuals with hypertension and cardiovascular disease most frequently reported sleeping four to six hours or seven to eight hours, while those with respiratory illness and autoimmune conditions showed no clear pattern in sleep duration. These findings indicate that while the length of time since diagnosis differs by illness type, sleep duration does not significantly vary across chronic disease categories in this sample.

Table 11 presents the association between current treatment type and two variables: duration of chronic illness and average sleep duration per night, using the chi-square test for categorical comparison. A statistically significant association was found between treatment type and duration of chronic illness (χ² = 21.4, p = 0.05). Participants receiving both medication and therapy (n = 130) were more frequently diagnosed one to three years ago (n = 48) or four to six years ago (n = 35), suggesting a trend toward combined treatment in mid-stage illness duration. Similarly, those undergoing therapy or counseling only (n = 127) had a large proportion within the one-to-three-year (n = 47) and four-to-six-year (n = 42) categories. In contrast, those receiving no treatment (n = 53) were more often diagnosed less than one year ago (n = 24), indicating that early-stage patients may be underdiagnosed or untreated.

**Table 11:** Descriptive statistics of demographic variables (type of current treatment, duration of chronic illness, and duration of sleep per night) f: frequency; %: percentage; p: level of significance; p-values calculated using the chi-square test; the significance level is set at p < 0.05

Variables	f	Less than 1 year	1-3 years	Duration of chronic illness (4-6 years)	7-10 years	More than 10 years	p	x^2^	Less than 4 hours	Duration of sleep per night (4-6 hours)	7-8 hours	More than 8 hours	p	x^2^
Current treatment type	-	-	-	-	-	-	0.05	21.4	-	-	-	-	0.698	6.41
Medication only	76	31	28	12	3	2	-	-	15	29	23	9	-	-
Therapy/counselling only	127	31	47	42	6	1	-	-	24	52	36	15	-	-
Both medication and therapy	130	35	48	35	11	1	-	-	28	41	47	14	-	-
None	53	24	13	10	5	1	-	-	7	18	23	5	-	-

In comparison, no significant association was found between treatment type and average sleep duration per night (χ² = 6.41, p = 0.698). Sleep patterns were relatively evenly distributed across treatment groups. For instance, those undergoing both medication and therapy primarily reported four to six hours (n = 41) or seven to eight hours (n = 47) of sleep, while participants in the “none” group also reported similar sleep patterns. This finding suggests that current treatment status does not significantly influence sleep duration among participants with chronic illness.

## Discussion

Our study evaluates the relationship between stress and fatigue severity in patients with chronic illnesses. The results depict a positive correlation between perceived stress and severity of fatigue, where higher levels of stress resulted in greater fatigue. Perceived stress is a significant predictor of fatigue severity scores. Perceived stress did not have any meaningful associations with alcohol use, types of chronic illnesses, or sleep duration. Our data showed nonsmokers having higher perceived stress than smokers. Although significant, this observation is not clinically meaningful. Higher fatigue levels were noted among smokers and those with short and longer sleep durations. However, fatigue severity had no significant relationship with alcohol consumption, nor did it have any meaningful variation based on the type of illness. A statistically significant association was observed between the type of treatment and duration of chronic illness, indicating that those in earlier stages of their illness may be underdiagnosed or untreated.

Previous studies have also reported a similar relationship between perceived stress and fatigue severity. A study on psoriasis patients by Yuksel et al. found that patients with depressive, cyclothymic, and anxious temperaments were more likely to have higher perceived stress and hence fatigue [15]. Another study on patients with systemic lupus erythematosus revealed that stress acts as a mediator between depression and fatigue [16]. Stress is not a collection of symptoms but is a result of an individual’s perception of life events. Chronic stress activates the HPA axis, leading to sustained cortisol release, which, over time, disrupts the body’s natural response to stress, resulting in fatigue, sleep disturbances, and cognitive impairments [16,17]. A study conducted on adults with myalgic encephalomyelitis concluded that stronger stress management skills are linked to reduced illness burden by decreasing emotional distress and, hence, fatigue, regardless of the severity of symptoms [18].

The results of our study did not show any significant association between perceived stress and sleep duration; however, it did reveal that long and short sleep durations resulted in increased severity of fatigue. In a previous study conducted on individuals with chronic pain, high stress was associated with a two times higher risk of shorter sleep duration and a 30% risk of longer sleep duration, as compared to individuals with lower stress [19]. As our study has established an association between perceived stress and fatigue severity, this may be one of the reasons why higher fatigue symptoms were observed in those with short and long durations of sleep. In another study on cardiovascular disease patients, increased levels of perceived stress were correlated with shorter sleep duration and increased fatigue. Substantial sleep debt is known to affect an individual’s mood and performance. Moreover, inflammatory cytokines such as TNF-alpha and IL-6 are fatigue-inducing cytokines that are inversely affected by sleep duration [20].

Increased fatigue levels were observed among smokers compared to nonsmokers. This outcome is similar to the literature online [21,22,23]. The oxidative stress that results from the introduction of free radicals into the body by smoking damages muscle fibers, increasing muscle fatigue. Furthermore, smoking reduces oxygen delivery to the muscles, hence impairing muscle function and increasing fatigue during activities [24].

This study has several limitations. A cross-sectional study means that we cannot confirm any direct connection between the levels of stress and fatigue in this dataset. Further studies should be done to investigate these relationships as they evolve. There is also the influence of recall bias and social desirability bias in response to the use of questionnaires, which can affect the study’s results. Although the given sample size is acceptable, including additional participants from a more diverse population would enhance the reliability and utility of the results for a broader audience.

The study’s sample, obtained from a few chosen healthcare facilities in Islamabad, may not be generalizable to other places because convenience sampling was used. Selection bias may also be present, as individuals who agree to participate may differ systematically from those who decline. Furthermore, potential confounding variables, such as the severity of illness, comorbid psychiatric conditions, medication use, or socioeconomic status, were not fully accounted for in the analysis and may have influenced the observed associations. Nevertheless, the study provides valuable insights into how psychological stress impacts fatigue in individuals living with chronic diseases, laying the groundwork for further research in this field.

## Conclusions

The findings of our study demonstrate a moderate positive correlation between the PSS and FSS (r = 0.481, p < 0.01), with stress being a significant predictor of fatigue. Severe fatigue levels were noted among smokers and those on the extreme ends of sleep duration. However, perceived stress and fatigue severity did not show any meaningful association with alcohol use, nor did they vary among types of chronic illnesses. Given fatigue's impact on quality of life and daily functioning, recognizing the role of stress is crucial for effectively managing chronic illnesses. Interventions that target stress reduction, such as cognitive behavioral therapy and structured coping strategies, can alleviate fatigue symptoms if introduced into the treatment regimens of patients. Longitudinal studies should be conducted on this topic to better understand the causal relationship between perceived stress and fatigue and to assess the long-term effects of stress-reduction interventions. Healthcare practitioners should be encouraged to monitor stress levels and incorporate psychosocial interventions as a part of treatment regimes in people with chronic illnesses.

## Appendices

Appendix 1

**Table 12:** Demographic information

Items	Responses
Age	-
Gender	-
Marital status	-
Educational level	-
Employment status	-

Appendix 2

**Table 13:** General health and lifestyle

Items	Responses
Type of chronic illness	-
Duration of chronic illness	-
Current treatment type	-
Do you smoke?	-
Do you consume alcohol?	-
Average sleep duration per night	-

Appendix 3

**Table 14:** Perceived Stress Scale (PSS) Developed by Levenstein et al. (1993), permission was obtained for use in this study [13].

Items	Responses
In the last month, how often have you been upset because of something that happened unexpectedly?	-
In the last month, how often have you felt that you were unable to control the important things in your life?	-
In the last month, how often have you felt nervous and stressed?	-
In the last month, how often have you felt confident about your ability to handle your personal problems?	-
In the last month, how often have you felt that things were going your way?	-
In the last month, how often have you found that you could not cope with all the things that you had to do?	-
In the last month, how often have you been able to control irritations in your life?	-
In the last month, how often have you felt that you were on top of things?	-
In the last month, how often have you been angered because of things that happened that were outside of your control?	-
In the last month, how often have you felt difficulties were piling up so high that you could not overcome them?	-

Appendix 4 

**Table 15:** Fatigue Severity Scale (FSS) Developed by Krupp et al. (1989), permission was obtained for use in this study [14].

Items	Responses
My motivation is lower when I am fatigued.	-
Exercise brings on my fatigue.	-
I am easily fatigued.	-
Fatigue interferes with my physical functioning.	-
Fatigue causes frequent problems for me.	-
My fatigue prevents sustained physical functioning.	-
Fatigue interferes with carrying out certain duties and responsibilities.	-
Fatigue is among my three most disabling symptoms.	-
Fatigue interferes with my work, family, or social life.	-
